# Patterns of sexual risk behavior among undergraduate university students in Ethiopia: a cross-sectional study

**Published:** 2012-06-18

**Authors:** Tariku Dingeta, Lemessa Oljira, Nega Assefa

**Affiliations:** 1 Haramaya University, Harar, Ethiopia

**Keywords:** Students, university, undergraduate, Ethiopia, Sexual risk behavior, youth, HIV/AIDS, sexual intercourse

## Abstract

**Introduction:**

As part of the young age bracket, undergraduate university students are exposed to a range of risky behaviors including HIV/AIDS. Given the paucity of data among the risk behaviors of African university students, this study was conducted to examine the sexual risk behaviors of this group in Ethiopia.

**Methods:**

A self-administered questionnaire was used to collect information on socio-demographic and sexual risk behavior characteristics among 1,286 undergraduate students at Haramaya University, Ethiopia from March to April, 2010. Multivariate logistic regression models were used to derive adjusted odds ratios (OR) and 95% confidence intervals (95% CI).

**Results:**

About 355 (28%; 95% CI 25.5-30.5) students reported to have had sexual intercourse at least once. More proportion of male students ever had sex compared to females (OR 4.8; 95% CI 3.4-6.8, p<0.001). One fifth (22.8%) of these students had their sexual debut after they joined university. About six percent of students with sexual experience reported having had intercourse with same-sex partners. Half of the males with sexual experience had intercourse with a commercial sex worker. About 60% of students reported to have used a condom rarely.

**Conclusion:**

Our findings indicate that there is a high level of sexual risk behavior among the study population. Significant proportion of students were sexually active, the majority started sexual intercourse before they joined university. We recommend awareness campaigns and interventions on sexual and reproductive health issues for high school and university students in Ethiopia.

## Introduction

HIV/AIDS is continuing to be a global challenge. Sub-Sahara Africa with an estimated 22.9 million people living with HIV in 2010 is the most affected part of the world [1]. Young people are among the most vulnerable groups; half of new infections in this region in the year 2009 occurred among those in the age range of 15 to 24 [1, 2]. The common, risky, sexual practices in this age group include early sexual intercourse, multiple sexual partners, unprotected sexual intercourse, engaging in sex with older partners and non-regular partners such as commercial sex workers [3–6]. Monitoring and changing the behavior of this vulnerable group is paramount in order to control the HIV pandemic [2, 7].

As part of the young age bracket, undergraduate university students are an important group exposed to a range of risky behaviors. The increased privacy afforded by living outside of their parents? home provides greater opportunity for sexual expression. Risky behavior among undergraduate students may be further worsened by the fact that they mostly live in campuses without boundaries or security; peer pressure; economic problems and lack of youth friendly recreational facilities [8, 9]. Particularly, risky behavior such as the consumption of alcohol, cigarette smoking, or the use of illicit drugs by adolescents have been shown to be associated with increased risks of sexual intercourse, multiple sexual partners and lower rates of condom use [3, 10–12].

Despite much research focusing on school going and out-of-school youth, little research has been done on the sexual risk behavior of university students in Ethiopia. This study aims to examine the sexual risk behavior of undergraduate students in Haramaya University, eastern Ethiopia.

## Methods

### Settings and study design

An institution based descriptive cross sectional study was conducted in Haramaya University which is one of the 21 governmental universities in Ethiopia. It is located in eastern Ethiopia around 513 kilometers from Addis Ababa, the capital city of Ethiopia. It enrolls students from all regions of the country and had around 14,000 in-campus students studying in 31 departments at the time of the study, March to April, 2010.

### Data collection

A total of 1286 students were included in the study. They were randomly selected in a proportional manner in which all departments and both sexes were represented. Selected students were then approached by trained data collectors. The randomly selected students were notified through the student representative and the department secretary. They were then provided orientation in their class rooms about the study, how they were selected, and confidentiality issues. Students that were willing to participate were provided with the questionnaire.

Due to the sensitive nature of the study and the educational background of the respondents, a privately self-administrated, structured questionnaire in the English language was used to obtain information. Almost all sections pertaining to the behavioral aspect were based on the Behavioral Surveillance Survey questionnaire [13] available online [14]. However, necessary modifications were made to suit the sample population by a panel convened by the National HIV/AIDS Prevention and Control Secretariat (HAPCO) of Ethiopia. The questionnaires were pre-tested for this study among Hawasa University students in southern Ethiopia and modifications were made afterward. Data were collected in two areas: socio demographics and sexual risk behaviors.

### Operational definition

## Data analysis

Data were entered using EPI Data Software version 3.1. They were then exported to Statistical Package for the Social Sciences software (IBM^®^ SPSS^®^ Statistics'IBM^®^ SPSS^®^ Statistics) version 15 for Windows for analysis. Descriptive statistics included frequencies and proportions. Associations were examined using chi-square tests and simultaneous entry multivariate logistic regression. Unadjusted and adjusted (AOR) odds ratios were used as indictors of the strength of association. The type I error rate was set at less than or equal to 0.05.

### Ethics statement

Since this is part of a bigger project on sexual risk behavior among university campuses in Ethiopia, ethical clearance was obtained from the Institutional Ethical Review Board (IERB) of Hawassa University, College of Health sciences through the recommendation of HAPCO. Written consent was obtained from each study participant in a form attached with the questionnaire. To ensure confidentiality and openness in reporting, anonymous questionnaires were provided to students along with an envelope. After administration, students sealed the questionnaires and returned them to the data collection coordinators. The consent forms we detached from the questionnaire by the students and placed in a box.

## Results

### Socio-demographic characteristics

A total of 1272 students responded to the questionnaires providing a response rate of 98.9%. About 67% (855) of the respondents were males, 33% (417) females. The mean (SD) age of the participants was 21.4 (1.3). All most all 1197 (97.5%) of the students reside in student accomodation facilities located inside the university campus (Table 1).

**Table 1:** Background characteristics of the study population at Haramaya University, 2010

Variables	Number	Percent
**Sex**		
Male	855	67.2
Female	417	32.8
**Religion**		
Orthodox	601	47.4
Catholic	26	2.1
Protestant	288	22.7
Muslim	304	24
Others	48	3.8
**Ethnic group**		
Oromo	662	52.6
Amhara	299	23.8
Tigray	68	5.4
Gurage	83	6.6
Others	146	11.6
**Academic year**		
Year one	492	38.9
Year two	357	28.2
Year three	389	30.7
Year four and five	28	2.2
**Income per month from your parents or relatives**		
< 100	351	29.8
101-200	404	34.3
201-300	237	20.1
> 300	185	15.7

### Sexual behaviors of participants

About 355 (28%; 95% CI 25.5-30.5) students reported to have had sexual intercourse. More male students had ever sex compared to females (OR 4.8; 95% CI 3.4-6.8,p < 0.001). The mean (SD) age at the first sexual intercourse was 17.54 (2.8) years; 17.5 (2.7) years for males and 17.76 (3.5) for females. Most of the students (271, 77.2%) who reported to have commenced sexual intercourse had their first sex with a girl or boy friend. Six (2%) male students reported having had first sexual intercourse with a commercial sex worker. Forty three (22.8%) reported to have had their first sexual intercourse after they joined university. Twenty two (6.4%) of the sexually active students reported to have practiced sex with the same sex partner from which 17 (5.7%) were males and 5 (11.4%) females. Five female students (12.2%) reported that they had been raped; one of them was raped after joining the university (Table 2).

**Table 2:** Sexual behavior of undergraduate students at Haramaya University, 2010

Variables	N	(%)
**Ever had sexual intercourse(n = 1249)**		
Yes	355	28.4
No	894	71.6
**At what school level did you first have sex**		
Primary school	34	18
Secondary school	112	59.25
University	43	22.75
**Sexual behaviour in the past 12 months**		
Ever had sexual intercourse in the past 12 months(n = 352)		
Yes	179	50.9
No	173	49.1
**In the past 12 months, how many sexual partners did you have sex with?(n = 179)**		
One only	119	66.5
Two or more	60	28.4
**In the past 12 months have you ever had sex with a person who is not your regular sexual partner(n = 179)**		
Yes	135	75.4
No	44	24.6
**How many of your sexual partners in the last 12 months were non-regular?(n = 135)**		
One only	96	71.1
Two or more	39	28.9
**How many times you had sex in the last 12 months with commercial sex partners? ( For males) (n = 79)**		
One only	57	72.2
Two only	14	17.7
Three or more	8	10.1
**Condom use**		
**Have you ever used condom?**		
Yes	220	64.1
No	123	35.9
**How frequently did you use condoms?**		
**Always**	39	20.4
**Occasionally**	37	19.4
**Rarely**	121	60.2
**Had sex without condom in the last 12 months**		
Yes	50	22.9
No	168	77.1
**Do you use condom when you had sex for the 1st time?**		
Yes	116	32.7
No	236	67.3
**Do you use condom in your last sexual encounter**		
Yes	174	49.4
No	178	50.6

About 179 (50.9%) students had sexual intercourse in the 12 months before the study period. Among these, 60 (33.5) had sex with two or more partners. More proportion of females (29, 67.4%) than males (150, 48.5%) had sex in the past 12 months (OR 2.2 (1.2-4.3),P 0.02). Twenty nine percent (60) of those that reported to have had sex in the past 12 months reported that they had more than one sexual partner. Out of the students sexually active during past 12 months about three in four (135, 75.4%) had sex with a non-regular partner. Sexually active male respondents were further asked about their sexual encounters with commercial sex workers (CSW) during the past 12 months. The results show that more than half (52.7%) ever had sexual intercourse with a CSW (Table 2).

### Condom use

Among students who reported to have ever had sexual intercourse, 220 (64.1%) had used a condom at least once. Less than half (116, 32.7%) had used condoms during their first sexual encounters. As shown in Table 2, condom use was high in most recent sexual relations (49.4%). Among those that ever used a condom, 39 (20.4%) used it consistently (Table 2).

### Predictors of risky sexual behaviors

A multivariate logistic regression model examining the relationship between gender and having multiple sexual partners and controlling for possible compounding by year of study, religion and substance use shows that male gender (OR 4.0; 95% CI 1.4-17.0) and attending night clubs (OR 2.0; 95% CI 1.1-7.0) were associated with having multiple sexual partners (Table 3).

**Table 3:** Multiple logistic regression analysis for association with having multiple sexual partners among the undergraduate students at Haramaya University, 2010

Variable	Crude OR (95% CI)	Adjusted OR (95% CI)	P-value
**Sex**			
Male	2.9 (1.3-6.3)	4.1 (1.4-17.0)	0.04
Female	1.00	1.0	
**Year of study**			
First year	0.9 (0.5-1.4)	1.1 (0.5-2.7)	0.91
Second year	0.9 (.6-1.2)	1.2 (0.5-2.9)	0.8
Third year and above	1.00	1.0	
**Religion**			
Orthodox	1.00	1.0	
Protestant	1 (0.5-1.6)	1.2 (0.5-3.0)	0.75
Muslim	0.9 (0.5-1.5)	1.4 (0.6-3.6)	0.44
Others	0.5 (0.2-1.23)	0.5 (0.12-1.6)	0.22
**Substance use**			
Yes	2 (1.3-3)	0.8 (0.3-3.8)	0.51
No	1.00	1.0	
**Ever watched pornographic films**			
Yes	1.7 (1.1-2.7)	1.5 (0.7-3.0)	0.33
No	1.00	1.0	
**Attend night clubs during the last three months**			
Yes	3.7 (2.1-6.4)	2.9 (1.1-7.0)	0.037
No	1.00	1.0	

## Discussion

The findings indicate that close to a third of the students had their first sexual encounter at a mean age of 17.5 years, half of these had sex in the past 12 months. About one fifth (22.8%) of the students who previously had sex reported to have had their first sexual encounter after they joined university. About six percent of students who ever had sex reported to have had intercourse with same-sex partners. Male gender was significantly associated with sexual debut (OR 4.8; 95% CI 3.4-6.8). Half of the males with sexual experience had intercourse with a commercial sex worker. About 60% of the students reported to have used a condom infrequently.

This study indicates a previous sex prevalence of about 28.4% among the sampled university students. This prevalence is higher than that reported in previous national survey from Ethiopia among school youth in which 9.9% were found to have had sexual experience [13, 15], this could probably be because of the higher age of university students. A greater proportion of Haramaya University students are sexually active compared to the 8% who had ever had sex among students from Huhan University in China [16], but much lower than the 39% and 76.8% report among undergraduate students in Nigeria and Nepal respectively [17, 18]. Differences among the studies reflect the sex education activities and cultural expectations of the population from which the samples were taken. But the figures highlight an important disparity between the expectations of preventing premarital sexual intercourse and the reality on the ground. Despite the differences in ever sexual intercourse, the age of first sexual intercourse was comparable among the studies, indicating that most students initiated sexual activity during early adolescence. These indicate the need for effective sex education for students in Ethiopia and elsewhere at an early age, probably in their high school years.

The study also showed that risky sexual behavior is common among the participants. Among the male students that ever had sex in the 12 months before the study period, a significant proportion (38%) had sexual intercourse with a commercial sex worker without using a condom. This is very high when we compare it with a study from Nepal on which more than a quarter of students (23%) had sexual intercourse with a prostitute [9]. This figure is also far higher when we compare it with undergraduate students from the University of Gondar in North West Ethiopia in which only 7.1% of the students had sexual contact with a prostitute [19]. More than 38% of those who had sexual intercourse with a prostitute in the past 12 months had not used a condom. This report is far different from the reports by authorities citing good risk perception and knowledge of mode of transmission of HIV/AIDS among youth in Ethiopia [12] at least not for university students. Of students who had engaged in sexual intercourse, about 24.5% of them reported having multiple partners. Compared with the reports from China and other countries like Nepal, the prevalence of multiple sex partner behavior among Haramaya university undergraduate students is relatively low [13, 16, 18].

Forced sex and homosexuality are considered a special category of high-risk sexual intercourse. Five (5/1272, 3.9%) female students reported being raped. Two of them were raped after they joined university; one of them was raped outside campus. This is a very saddening situation, also further investigated by Haile-Meskel and Gebre-Medhin among female samples in the same university. They reported that about 3% of females experienced rape in their life time, 1.8% in the last 12 months, resulting in pregnancy in 14% of cases. The main risk factors for rape were having a boy friend (OR 3.59; 95% CI not available) and living off campus (OR 2.83; 95% CI 1.08-7.44). Furthermore they reported that 27.8% experienced uninvited sexual overtures such as verbal jokes including direct solicitation for sexual intercourse; and 19.3% encountered unwelcome touching of their bodies [20]. This calls upon legal and university authorities to work more on protecting students and creating awareness about the existence of risk of rape.

Homosexual sex is considered a taboo and illegal in Ethiopia like many other African countries. This study revealed that 22 (6.4%) of the sexually active students reported to have practiced homosexual sex. This figure is similar to the 7.6 percent reported in an unpublished study among students of Dire Dawa University, Ethiopia [21]. This indicates that despite the cultural taboo and illegality of homosexuality in Ethiopia and other African countries, there are sections of youth practicing it. It is clear that this group is at a disadvantage when it comes to reducing risks due to the sensitive nature of their act and the possibility of promiscuity. For instance the study among Dire Dawa University students found a high risk of HIV infection among this group compared to heterosexuals (OR 10.5; 95% CI 1.9-56.4) [21].

Overall prevalence of ever use of condom in sexual relations in this sample of undergraduate students was 64.1%. Findings from a behavioral surveys and DHS Ethiopia in 2005 differed from our findings in relation to ever use of condom prevalence. About 43.1% and 45.9% of the sexually active youth samples reported ever use of condoms in the National Behavioral Surveillance Survey and DHS 2005 respectively [13, 15]. In this study, among the sexually active students that ever used a condom less than quarter (20.4%) of them had used condoms consistently. The findings show that despite the expectation of prevention consciousness among university students, the prevalence of consistent use of condom is very low compared to findings elsewhere [3, 12]. However, this result is better when compared to the study among Gondar university students in Ethiopia where the percentage was a mere 6.4% according to a brief communication by Fitaw and Worku in 2005 [19]. This shows a similar and persistently low use of condoms among university students in Ethiopia. If consistent condom use remains low, vulnerable sexual networks and practices will continue among the students, allowing for the faster spread of sexually transmitted diseases and HIV. These findings therefore highlight a very high vulnerability among university students and calls for more rigorous prevention and awareness creation campaigns.

This study examined sexual risk behavior and multiple sex partner behavior and its risk factors among under graduate students in an Ethiopian university. Our findings indicate that there is an alarming level of sexually risky behavior among the study population. Significant proportion of students were sexually active, the majority started sexual intercourse before they joined university. Out of those who had sex in the past twelve months, more than 40% of students had sex with multiple sexual partners including prostitutes. There is limited use of condoms and those that use them do not do so consistently. Similarly, condom use at first sexual intercourse was also very low. This indicates that the university students are exposed to health hazards through their risky sexual behavior.

Several factors are involved in the process of the decision for sexual experimentation among youth. Sensation seeking, impulsivity, curiosity, use of substances such as alcohol, and lack of self-regulation seem to contribute to the problem [17, 22]. However, females are put at a higher risk by more complex factors such as sexual intercourse for financial reasons and rape [17, 20]. Therefore, interventions at tackling sexual risk behavior need to also focus on these factors.

## Conclusion

We recommend that concerted efforts be expended in order to address sexual and reproductive health problems of university students. These interventions should target both high schools and universities to dissuade risky sexual behavior and render sexual intercourse safe. The interventions could include comprehensive sexual and reproductive health education on issues such as sexual education, safe sex and sexually transmitted infections.
